# Development and Management of Avoidant/Restrictive Food Intake Disorder and Food Neophobia in Pediatric Patients with Food Allergy: A Comprehensive Review

**DOI:** 10.3390/nu16173034

**Published:** 2024-09-08

**Authors:** Rita Nocerino, Caterina Mercuri, Vincenzo Bosco, Vincenza Giordano, Silvio Simeone, Assunta Guillari, Teresa Rea

**Affiliations:** 1 Department of Translational Medical Science, University of Naples Federico II, 80131 Naples, Italy; rita.nocerino@unina.it (R.N.); assunta.guillari@unina.it (A.G.); 2 ImmunoNutritionLab at CEINGE Advanced Biotechnologies, University of Naples Federico II, 80131 Naples, Italy; 3 Department of Biomedicine and Prevention, University of Rome “Tor Vergata”, 00133 Rome, Italy; 4 Department of Clinical and Experimental Medicine, University of Catanzaro MagnaGraecia, 88100 Catanzaro, Italy; c.mercuri@unicz.it; 5 Department of Medical and Surgical Sciences, University Hospital Mater Domini, Magna Graecia University, 88100 Catanzaro, Italy; vincenzo.bosco@unicz.it; 6 Cardarelli Hospital, 80131 Naples, Italy; enza-giordano@hotmail.it; 7 Department of Public Health, University of Naples “Federico II”, 80131 Naples, Italy; teresa.rea@unina.it

**Keywords:** food allergy, pediatrics, eating disorders, Cognitive Behavioral Therapy, nutritional deficiencies, psychosocial impact, nursing

## Abstract

Avoidant/Restrictive Food Intake Disorder (ARFID) and food neophobia present significant challenges in pediatric healthcare, particularly among children with food allergies (FAs). These eating disorders, characterized by the persistent avoidance or restriction of food, can lead to severe nutritional deficiencies and psychosocial impairments. The presence of FAs further complicates these eating behaviors, as the fear of allergic reactions exacerbates avoidance and restrictive patterns. This comprehensive review synthesizes current knowledge on ARFID and food neophobia, focusing on their definitions, characteristics, and the unique challenges they present in the context of FAs. The review explores the critical role of healthcare professionals, especially nurses, in integrating psychological and clinical care to improve outcomes for affected children. A multidisciplinary approach, including Cognitive Behavioral Therapy (CBT) and Family-Based Therapy (FBT), is emphasized as essential in addressing the complex needs of these patients. The review also highlights the need for standardized treatment protocols and further research on the long-term outcomes of these disorders, aiming to enhance therapeutic strategies and family support systems. Effective management of ARFID and food neophobia in the context of FAs requires a holistic and integrated approach to mitigate the profound impacts on a child’s growth, development, and overall well-being.

## 1. Introduction

Avoidant/Restrictive Food Intake Disorder (ARFID) and food neophobia represent significant challenges in pediatric healthcare, particularly impacting children with food allergies (FAs) [1,2,3,4]. These eating behaviors are closely correlated with the presence of FAs, as children with FAs are at increased risk of developing avoidant behaviors toward food and feeding due to fear of allergic reactions [5,6,7]. Both ARFID and food neophobia are significant concerns due to their biopsychosocial consequences, including anxiety, which can profoundly impact daily activities and social interactions [8,9,10,11,12]. Children with ARFID may experience significant anxiety and distress related to eating, which often extends to other areas of their lives [10]. This condition profoundly influences the quality of life of both the children and their parents, affecting the child’s development and potentially leading to long-term health issues such as lower bone density and shorter stature, as recently discussed by Proctor et al. [13,14,15,16].

ARFID, first recognized as a distinct diagnostic category in 2013, is characterized by the persistent avoidance or restriction of food intake, leading to nutritional deficiencies, weight loss, and psychosocial impairments without concerns about body weight or shape that characterize other eating disorders [17,18]. The behavior toward food in children with ARFID is often shaped by a complex interplay of psychological factors, including anxiety about eating and the potential for allergic reactions [1,2,19].

Similarly, food neophobia, introduced by Pliner and Hobden in 1992 [20], defined as the fear or refusal to try new foods, commonly occurs in early childhood and can result in limited dietary variety [8,9]. Parental anxiety plays a significant role in the development of both ARFID and food neophobia, as parents may report higher feeding and emotional disturbances in children with these conditions [21,22,23]. In children with FAs, food neophobia may be more pronounced due to previous negative experiences with food and heightened anxiety related to the possibility of an allergic reaction [8,24,25,26].

The etiology of both ARFID and food neophobia is multifactorial, with FAs identified as one of the contributing factors [5,9,27,28,29]. Children with FAs may develop anxiety and fear related to eating due to the risk of allergic reactions, which can exacerbate avoidance behaviors and lead to or worsen ARFID and food neophobia [1,2,30]. Other contributing factors include genetic predispositions, sensory sensitivities, and negative past experiences with food [16,22].

Moreover, it is important to recognize that both ARFID and food neophobia may be linked to FAs through shared mechanisms of anxiety and avoidance behavior, as children with FAs often develop a heightened sense of fear toward consuming new or previously tolerated foods due to the potential risk of allergic reactions [7,31,32].These conditions can lead to significant social impairments and contribute to psychological distress, further complicating the child’s overall well-being and development [23,33].

Both conditions can have profound implications for a child’s growth, development, and overall well-being, particularly when complicated by the presence of FAs [1].

The intersection of ARFID, food neophobia, and FAs presents a complex clinical picture that heightens the risk of nutritional deficiencies and impacts growth and development [2,21]. Parental anxiety and feeding practices also play a role in the development and maintenance of these eating behaviors, further affecting the child’s quality of life [10,34].

Given the rising prevalence up to 22% of these conditions and their potential persistence into adulthood causing medical, psychological, and social issues, a comprehensive understanding of their development, management, and impact on pediatric patients with FAs is essential [10,34,35,36,37,38,39,40,41,42].

This review aims to synthesize current knowledge on ARFID and food neophobia, with a specific focus on their correlations with behavior toward food and feeding in the context of FAs.

Furthermore, it will explore the critical role of healthcare professionals, particularly nurses, in integrating psychological and clinical care, to improve outcomes for affected children.

## 2. Materials and Methods

### 2.1. Objectives

This comprehensive review investigates the literature on ARFID and food neophobia in the pediatric population (0 to 18 years) with FAs. The aim is to provide a thorough understanding of the clinical and psychological aspects of these disorders, explore potential treatments, identify gaps in the current literature, and suggest areas for future research.

### 2.2. Search Strategy

The literature search was conducted using the CINAHL Complete, PubMed, and Scopus databases. Specific relevant keywords such as ‘food allergy’, ‘food allergies’, ‘ARFID’, ‘avoidant restrictive food intake disorder’, ‘selective eating disorder’, ‘selective feeding disorder’, ‘food neophobia’, ‘children’, ‘adolescents’, ‘nurse’, and ‘nursing’ were employed to identify eligible articles.

To include as many pertinent articles as possible, two search strings were formulated:

1. (food allergy OR food allergies) AND (ARFID OR avoidant restrictive food intake disorder OR selective eating disorder OR selective food intake disorder OR neophobia) AND (children OR adolescents OR youth OR child OR teenager) AND (nurse OR nurses OR nursing)
2. (food allergy OR food allergies) AND (ARFID OR avoidant restrictive food intake disorder OR selective eating disorder OR selective food intake disorder OR neophobia) AND (children OR adolescents OR youth OR child OR teenager)

The search strings were developed collaboratively by the authors, who then retrieved and evaluated relevant articles by reading the full texts.

### 2.3. Inclusion and Exclusion Criteria

#### 2.3.1. Inclusion Criteria

(a) Type of Studies: All types of studies, including systematic reviews, randomized controlled trials (RCTs), observational studies, and case reports.
(b) Language: Articles published in English or Italian.
(c) Participants: Studies involving participants diagnosed with FA and the subsequent development of ARFID or food neophobia.

#### 2.3.2. Exclusion Criteria

(a) Non-peer-reviewed articles, conference abstracts, and letters to the editor.
(b) Articles published in languages other than English or Italian.
(c) Studies published outside the 15-year period.

#### 2.3.3. Selection Process

**Figure 1:**
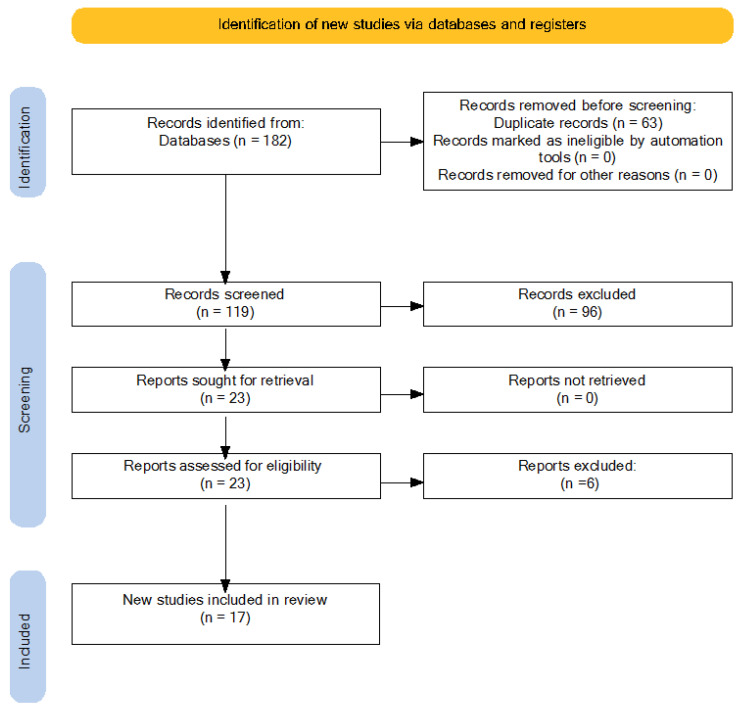
PRISMA diagram.

The review was conducted between May 2024 and July 2024. The search strategy identified 182 articles. After removing 63 duplicates, 119 articles remained for title screening. This step was performed independently by each author to ensure objectivity. Disagreements were resolved through discussion and consensus. After title screening, 23 articles were considered for full-text evaluation. Full-text reading was also carried out independently by the authors, leading to the final selection of 17 articles. Figure 1 reports the PRISMA diagram illustrating the selection process. The authors achieved a 100% agreement on the inclusion and exclusion of articles after a discussion in which individual articles were evaluated according to the inclusion criteria.

#### 2.3.4. Data Analysis

Data from the selected studies were grouped into thematic categories to describe psychological and behavioral aspects, clinical aspects, interventions and treatments, and the role of health professionals. The thematic analysis followed a structured approach involving coding, identification of themes, and synthesis of findings [43]. Key interconnections between variables such as food-related anxiety and psychiatric comorbidities were identified, to provide a deeper understanding of the development of ARFID and food neophobia in children with FA.

## 3. Results and Discussion

### 3.1. Food Allergy, ARFID, and Neophobia: Definitions, Etiology, and Clinical Aspects

FA is a prevalent chronic condition in children, arising from a disruption of immune tolerance to dietary antigens [44]. The prevalence, persistence, and severity of FA are increasing, leading to significant negative impacts on the health-related quality of life for both patients and their families, as well as escalating costs associated with its management [45]. In addition to the direct physical effects, FA can also affect social and psychological well-being [46], with the risk of developing restricted food preferences in children and altering their taste and sensory perception [2]. Although research in this area is still limited, it has been found that children with FA may develop maladaptive eating behaviors such as food refusal, tantrums, and reduced food intake in an attempt to avoid pain, discomfort, or anxiety [7].

These behaviors are not limited to the avoidance of allergens but may extend to normally tolerated foods [39]. The causes of such avoidance may include lack of appeal of the food, unpleasant sensory properties or fear of negative consequences, often exacerbated by genetic and psychosocial predispositions [28].

These factors may contribute to the development of ARFID and food neophobia, complex and multifactorial conditions that negatively impact eating behavior and psychological well-being [47]. The presence of FAs can exacerbate avoidant behaviors toward food, leading to more severe cases of ARFID and food neophobia [2,7,26,30,31,48].

ARFID is a relatively recent feeding disorder, first included as a diagnostic category in 2013 [49], and it is also recognized in the International Classification of Diseases, Eleventh Revision (ICD-11). It is characterized by persistent avoidance or restriction of food intake, leading to significant nutritional deficiencies, weight loss, and psychosocial impairment, without concerns related to body weight or shape [17,18]. Its etiology is complex and includes factors such as sensory sensitivities, traumatic experiences related to eating (e.g., choking, vomiting), comorbid psychological conditions, and medical issues like FAs [16,47,49]. FAs can contribute to ARFID development by instilling fear and anxiety around eating, leading children to avoid certain foods or entire food groups to prevent adverse reactions [2,30].

Food neophobia refers to the fear or refusal to try new or unfamiliar foods, typically emerging in early childhood [7,8]. Etiological factors include evolutionary protective mechanisms, genetic predispositions, and learned behaviors influenced by family and cultural practices [24,50]. In children with FAs, food neophobia may be intensified due to negative experiences with certain foods and heightened anxiety about potential allergic reactions, further restricting dietary variety and nutritional intake [26,48].

Both ARFID and food neophobia can have significant health implications, including impaired growth and development, nutritional deficiencies, and reduced quality of life [10,11]. The prevalence of these conditions varies, with some studies reporting rates ranging from 5% to 22.5% for ARFID in children and adolescents, influenced by factors such as age, geography, and diagnostic criteria used [10,34,35,36,38,39]. Understanding the etiological role of FAs in these disorders is crucial for effective diagnosis and intervention, as the management of underlying food allergies can be integral to addressing the associated feeding difficulties [27,51].

Early identification and comprehensive assessment are essential in managing ARFID and food neophobia, particularly when FAs are involved. Diagnostic tools such as the Eating Disorders Assessment for DSM-5 (EDA-5) and the Children Food Neophobia Scale (CFNS) can aid in accurately diagnosing and differentiating these conditions [52,53]. A thorough clinical evaluation should consider medical history, including any known FAs, psychological factors, and environmental influences to develop an effective, individualized treatment plan [52,54].

### 3.2. ARFID and Food Neophobia in the Context of Food Allergy

Both ARFID and food neophobia can be exacerbated by the presence of FAs, as the fear of allergic reactions can increase anxiety and food refusal behaviors [1,2,19]. The correlation between FAs and avoidant behaviors toward food and feeding is particularly pronounced in pediatric populations [2,7]. Children with FAs often develop a heightened sensitivity to food-related cues due to their experiences with allergic reactions [3,4] which can manifest in various ways, including avoidance of certain foods and mealtime anxiety [2,55], leading to the development of ARFID or exacerbating existing food neophobia [27,51].

In children with FAs, who already have restricted diets due to the need to avoid allergenic foods, the fear of trying new foods can further restrict dietary variety, resulting in a limited diet that lacks essential nutrients [27,51]. The fear of adverse reactions can cause children to become overly sensitive to food-related cues, intensifying their fear of trying new foods and resulting in restrictive eating behaviors [2]. Patients with ARFID typically wish to gain weight but refrain from eating for various reasons, such as the sensory properties of food, fear of potential negative outcomes from eating (e.g., vomiting, reflux, choking), or a general lack of interest in food [56,57].

In addition, the emotional response of parents to their child’s allergic reactions or at mealtimes also plays a crucial role in shaping the child’s eating behaviors, making them more fearful or anxious about eating, further limiting the child’s dietary variety [58]. Studies have highlighted that adverse reactions to foods are a significant concern for parents and can impact the family’s quality of life. Many parents report high levels of stress and anxiety related to managing their child’s FAs, which can influence the child’s eating behaviors and contribute to the development of ARFID [59]. Research indicates that anxiety symptoms in children with FAs are driven by perceptions of risk and the burden of managing their allergies, rather than their medical history alone [9]. This anxiety can extend beyond food-related situations, contributing to generalized anxiety and social phobia [60].

The recent National Birth Cohort study further supports that higher parental stress, especially related to specific allergens like hen’s egg, is linked with increased behavioral issues in children, potentially leading to neophobia and ARFID [61]. Furthermore, children with FAs often exhibit a broad range of gastrointestinal issues, which can compound their eating difficulties and contribute to the development of ARFID [62]. If not addressed, these conditions can significantly affect children’s growth and development, especially in the presence of FAs [1]. FAs are a substantial public health concern, affecting up to 11% of the pediatric population [45,55,63,64]. In pediatric subjects with FAs, the prevalence of concomitant eating disorders ranges widely from 8% to 62.9% [65,66].

The intersection of ARFID, food neophobia, and FAs presents unique challenges. Children with FAs are already at risk for nutritional deficiencies due to the necessary avoidance of allergenic foods [2]. The presence of ARFID and food neophobia can exacerbate these challenges, leading to more severe nutritional deficits and impacting growth and development [16]. Additionally, these children frequently exhibit anxiety disorders, including generalized anxiety, separation anxiety, and panic disorder, which further complicate their eating behaviors and overall health [10,37,67].

Understanding the complex relationship between ARFID, food neophobia, and FAs is essential for developing effective interventions. This includes addressing both the medical management of allergies and the psychological support needed to reduce food-related anxiety and improve dietary intake [61,62,65,66]. Effective management requires a multidisciplinary approach that integrates medical, nursing, psychological, and nutritional care, with a focus on reducing food-related anxiety and increasing dietary variety [10,27,60,67].

**Figure 2:**
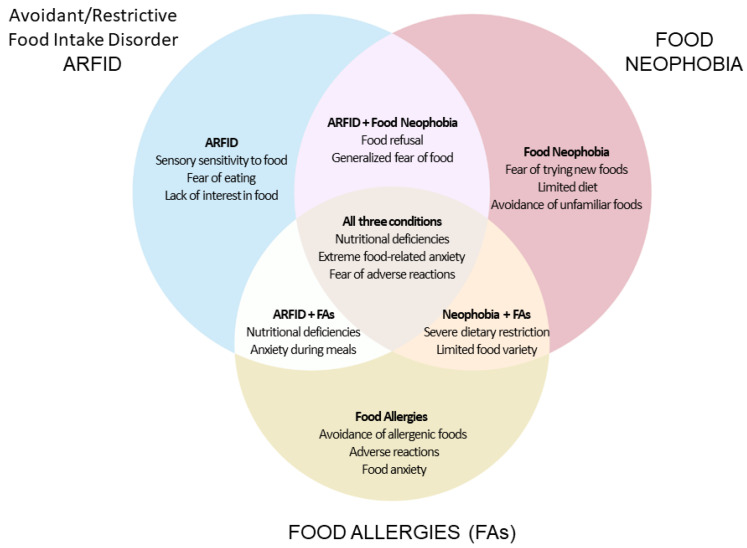
VENN diagram.

Figure 2 depicts a Venn diagram that illustrates the relationships and overlaps between ARFID, food neophobia, and FAs. This diagram helps to visualize how these conditions intersect, contributing to complex food-related challenges in affected individuals.

### 3.3. Interventions and Treatments: Comprehensive Approach

A comprehensive approach to managing ARFID and food neophobia involves integrating nursing, psychological, medical, and nutritional care through a multidisciplinary team. Given the complexity of the disorder, it is essential that a team of specialists is involved in its diagnosis and management, working together to develop a personalized treatment plan that takes into account the patient’s age and the severity of the disorder [32,68,69,70].

This strategy ensures that all aspects of the disorders, including nutritional deficiencies, psychological distress, and behavioral issues, are addressed [28,68]. In children with FAs, treatment plans must also incorporate strategies to manage the underlying allergies, which are often a central factor in the development and maintenance of ARFID and food neophobia [30,34].

A critical component of this approach is Cognitive Behavioral Therapy (CBT), which is particularly effective in treating ARFID by targeting avoidance behaviors and fears associated with food intake, thereby improving eating behaviors [71]. CBT for ARFID (CBT-AR) is a flexible, modular treatment typically conducted over 20–30 sessions. It includes four stages: psychoeducation and early change, treatment planning, addressing maintaining mechanisms, and relapse prevention. The therapy’s core components—food exposure, psychoeducation, anxiety management, and family involvement—help patients gradually confront and reduce food-related fears [72]. For pediatric patients with FAs, CBT may include components specifically designed to address the anxiety and fear related to allergic reactions, making it a tailored approach for this population [72,73].

The success of managing ARFID and food neophobia, especially in pediatric patients, hinges on a multidisciplinary team that often includes pediatricians, nurses, dietitians, psychologists, and other specialists. The integrated ‘whole team’ approach that characterizes specialized services, with strong leadership and a consistent treatment philosophy, appears to be more important for outcomes than the specific skills of individual practitioners working in separate settings [74].

These professionals work collaboratively to provide comprehensive care, with regular interdisciplinary meetings ensuring consistent updates and necessary adjustments to treatment plans. This team-based approach allows for tailored interventions that meet the unique needs of each patient [73]. Nurses play a crucial role in early identification, routine health assessments, and ongoing monitoring, while dietitians develop and monitor nutritional plans to ensure patients receive adequate nutrition despite their restrictive eating patterns. Pediatricians oversee the overall health and development of the child, addressing any medical concerns that arise, while psychologists provide essential support through interventions like CBT [73].

Family involvement is essential in the treatment process, as family dynamics can significantly impact a child’s eating behaviors, particularly for children with FAs, where parental anxiety and feeding practices significantly impact the child’s eating behaviors [22,34,70].

Active participation of parents or guardians in goal setting and treatment planning is essential, along with education and support to help them understand the disorder and support their child’s recovery at home [70]. In fact, family-based therapy (FBT) has shown promising results in treating ARFID and food neophobia, particularly in children. FBT focuses on empowering parents to take an active role in managing their child’s eating behaviors, which is crucial for overcoming the challenges associated with ARFID. Studies have demonstrated that FBT, when adapted for ARFID, significantly improves parental self-efficacy, leading to better outcomes in children’s eating habits and nutritional status. For instance, a randomized clinical trial found that children receiving FBT and Psychoeducational Motivation Therapy (PMT) exhibited notable improvements in weight gain and a reduction in food avoidance behaviors compared to those in other treatment groups [75]. The PMT can effectively improve motivation and reduce food-related anxiety, leading to positive changes in eating behaviors, even in cases where traditional behavioral interventions alone have been insufficient. Integrating cognitive behavioral strategies within FBT has been effective in increasing dietary variety and reducing food-related anxiety, making it a comprehensive approach for addressing food neophobia [76].

Given the lack of standardized management protocols, there is a critical need for developing individualized treatment plans that consider the patient’s age, developmental stage, the presence of FAs, and the severity of the disorder [77]. Pharmacological interventions, such as appetite stimulants and medications for managing co-occurring anxiety, may also be necessary as part of a comprehensive treatment plan [78,79]. Therefore, multidisciplinary treatment approaches have shown promising results in improving outcomes for ARFID patients. Recent studies, such as those conducted by Murphy and Zlomke (2016), Lenz et al. (2018), and Spettigue et al. (2018), support the effectiveness of this approach [80,81,82]. A randomized clinical trial showed that a day treatment program with intensive, portable interventions delivered by a multidisciplinary team led to significantly greater improvements, suggesting that a collaborative approach can effectively address chronic food refusal [69].

Multidisciplinary treatment approaches have shown promising results in improving outcomes for ARFID patients. Parents play a crucial role in the management of eating disorders. They need to be actively involved in treatment [83]. However, despite their importance, they often receive little support. Parents are often the first to recognize the disorder in their children and their involvement is essential, but they are not always adequately supported [24,83].

It is important to recognize the needs of parents and to encourage close collaboration between them and healthcare providers. Both need up-to-date information and ongoing support to manage the emotional burden and improve the management of the disorder [77,83,84].

A systematic review emphasized the need for well-defined protocols and training programs to ensure consistency and effectiveness in care delivery. It also highlighted the importance of nutritional rehabilitation as a critical component of treatment, recommending oral feeding as the preferred method and enteral feeding as a last resort for severely affected patients [79]. Additionally, studies have demonstrated the effectiveness of exposure-based interventions and CBT in reducing food neophobia and improving dietary intake, with evidence supporting their application in children, adolescents, and even individuals with intellectual disabilities and anxiety [85].

Nurses play a critical role as counselors in CBT, providing essential support to patients dealing with various psychological and physical conditions. For instance, Niesen et al. (2018) demonstrated that nurse-led CBT significantly reduces functional abdominal pain and enhances self-management. Similarly, Lee et al. (2011) found that nurse-led CBT reduces fatigue and improves quality of life in breast cancer patients undergoing radiotherapy [86,87]. Thus, evidence highlights the effectiveness of nurse-led CBT interventions in improving patient self-management and overall quality of life.

**Figure 3:**
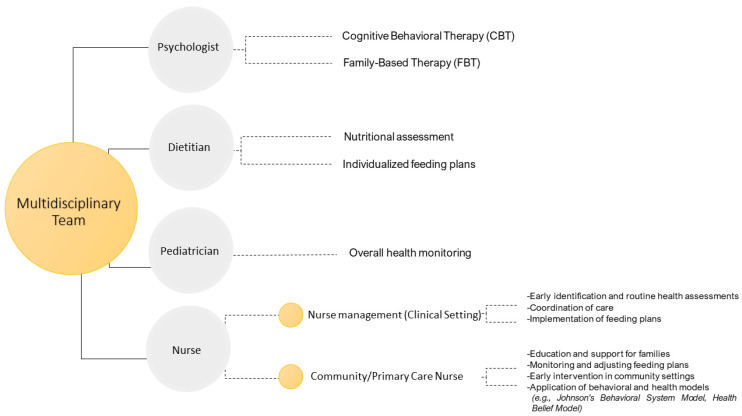
Multidisciplinary team approach to ARFID and food neophobia treatment.

The diagram in Figure 3 outlines the roles of a multidisciplinary team in the treatment of ARFID and food neophobia. This team-based approach ensures comprehensive care, addressing both the medical, nursing, and psychological needs of individuals with ARFID and food neophobia.

### 3.4. Role of Nurses in Managing ARFID and Food Neophobia

Nurses could play a critical role in the early identification and routine health assessments of pediatric patients with ARFID and food neophobia, particularly in pediatric patients with FAs. They are often the first healthcare providers to notice signs and symptoms of ARFID, such as significant weight loss, nutritional deficiencies, and psychosocial impairments. Regular growth monitoring and nutritional assessments are essential components of routine health check-ups, enabling early detection and intervention [88]. In cases involving FAs, nurses also monitor the management of allergies, ensuring that dietary interventions are safe and effective [27].

Nurses collaborate with dietitians to develop and implement individualized nutrition plans that address both the specific needs related to ARFID or food neophobia and the dietary restrictions imposed by FAs [79]. These plans often include strategies like food chaining, which involves gradually introducing new foods in a way that feels safe to the child. Nurses also monitor the child’s progress, adjust the care plan as needed, and ensure that the child receives adequate nutrition to support growth and development [27].

A systematic review highlighted the importance of a multidisciplinary approach involving pediatric gastroenterologists, nutritionists, neuropsychiatrists, and psychologists [79]. Nurses play a central role in this team by coordinating care, ensuring that all aspects of the child’s health are addressed. Their role is essential because they act as intermediaries between the patient and the entire healthcare team [89]. They facilitate communication between different team members and ensure that all diagnostic and therapeutic information is shared and understood [90]. This central role in coordination is crucial because it helps ensure that treatment is integrated and consistent, enabling more effective management of eating disorders [91].

This holistic approach is essential for managing the complex needs of children with ARFID [79].

Education and support for families are vital components of managing ARFID and food neophobia [92,93]. In fact, community nurses have a vital role to play as their role goes beyond care and includes relationship, support, and the application of behavioral and health theory [93].

Nurses provide education and support to families, helping them manage food-related anxiety and improve the child’s eating behaviors at home. This includes educating parents about the nature of ARFID, its potential impact on their child’s health, and effective strategies for encouraging healthy eating behaviors. They are uniquely positioned to offer ongoing emotional and psychological support, which is essential for maintaining a therapeutic environment in both clinical and home settings [93].

Community nurses play a significant role in improving caregivers’ abilities to care for children with ARFID. They help to develop effective strategies to improve children’s eating habits. This is crucial as many caregivers may lack the skills to deal with food selectivity and nutritional difficulties [69,94,95]. Through education, nurses provide tools and knowledge to help caregivers better manage such conditions [93].

They use models like Johnson’s Behavioral System Model (JBSM) and the Health Belief Model (HBM) to guide their interventions, helping parents develop constructive behaviors and build confidence in managing their child’s condition [93]. By applying these models, nurses can tailor interventions to meet the unique needs of each family, which is particularly important in managing the psychosocial aspects of ARFID.

A study by De Toro et al. (2021) emphasized the importance of a multidisciplinary approach and highlighted the role of pediatricians and nurses in diagnosing ARFID [96]. Nurses are essential not only in diagnosis but also in providing continuous support that integrates psychological, nutritional, and medical care, ensuring that families receive comprehensive care [96].

A recent study by Oktarina et al. (2023) demonstrated that health education interventions for mothers significantly improved their ability to manage toddlers with ARFID [92]. This study underscores the importance of community health nursing in providing targeted education and support to caregivers.

In addition to education, community nurses play a key role in meal planning and nutritional monitoring, intervening early to prevent complications such as weight loss and nutritional deficiencies. This early intervention is essential to prevent eating difficulties from developing into more serious problems [70,93].

Furthermore, nurses help to reduce the stress and emotional burden on careers by providing a supportive environment that is essential for the management of the psychological and social challenges associated with ARFID [97]. Their ability to offer both psychological and social support is critical for the overall well-being of families [89].

Moreover, school nurses have a unique role in managing children with ARFID and food neophobia within the school environment, particularly for children with FAs. They can implement individualized health plans, monitor dietary intake, and provide ongoing support and education to both children and their families. This is particularly important for children with additional needs, such as those with ASD, who may exhibit more pronounced food selectivity [98]. Table 1 summarizes strategies used by nurses to support families in managing ARFID and food-related anxiety.

## 4. Strengths and Limitations

This review offers a thorough and comprehensive synthesis of the existing literature on ARFID, food neophobia, and FAs in pediatric populations, highlighting several strengths. A key strength lies in its multidisciplinary approach, which integrates psychological, nutritional, and medical perspectives to provide a holistic understanding of these conditions and their impact on children’s health and development. The review emphasizes the importance of collaboration among pediatricians, psychologists, dietitians, and nurses in managing these eating disorders, underscoring the crucial role of nurses in early identification and intervention, as well as their support throughout the therapeutic process.

In addition to this, the review offers a detailed examination of effective therapies, such as CBT and FBT, both of which have been shown to be beneficial in improving eating behaviors and reducing anxiety associated with these disorders. This focus on evidence-based treatments presents practical solutions that can significantly enhance the quality of life for patients and their families while addressing the psychological impact of ARFID and food neophobia.

However, this review also has some limitations that need to be acknowledged. It is constrained by the limited availability of longitudinal studies, which restricts the ability to draw definitive conclusions about the long-term outcomes of ARFID and food neophobia in the context of FAs. The reliance on studies published only in English and Italian may introduce language bias, potentially excluding relevant research published in other languages. Additionally, the exclusion of non-peer-reviewed articles, while ensuring the quality of the review, might have led to the omission of emerging evidence in this rapidly evolving field. The lack of standardized treatment protocols for ARFID and food neophobia is another significant limitation, as it can result in inconsistent treatment approaches and variable outcomes.

Overall, while this review provides valuable insights and a well-structured analysis of ARFID and food neophobia, the identified gaps highlight the necessity for further research. There is a particular need for studies that explore the long-term effects and efficacy of multidisciplinary interventions, as well as the development of standardized protocols to ensure more consistent and effective management of these complex eating disorders in children with FAs.

## 5. Conclusions

This comprehensive review highlights the intricate interplay between ARFID, food neophobia, and FAs in pediatric patients, underscoring the significant challenges they pose to healthcare providers and families alike. The findings emphasize the critical need for early identification and multidisciplinary intervention to mitigate the profound impacts these conditions can have on a child’s growth, development, and overall well-being.

The role of nurses in these teams is central and multifaceted, encompassing early identification, care coordination, direct patient support, and education for families.

Effective management of ARFID and food neophobia, particularly in the context of FAs, requires an integrated approach that combines psychological, nutritional, and medical care, with a central role for nurses in both clinical and community settings. The review also identifies key gaps in current literature, including the need for standardized treatment protocols and more extensive research on the long-term outcomes of these disorders. Future efforts should focus on refining therapeutic strategies, enhancing family support systems, and expanding the role of healthcare professionals in addressing these complex and multifaceted conditions to improve the quality of life for affected children and their families.

## Figures and Tables

**Table 1:** Strategies used by nurses to support families in managing ARFID and food-related anxiety.

Intervention	Strategies	Reference
**Education and Empowerment**	**Providing Comprehensive Education:** Nurses educate families about ARFID, explaining its symptoms, causes, and potential impacts. By covering both the psychological and physical aspects of the disorder, they help families understand the importance of addressing these issues together.	Lee & Wang, 2016 [99]
	**Teaching Anxiety Management Techniques:** Nurses train parents and children in managing anxiety related to food intake, such as through deep breathing exercises, mindfulness, and relaxation techniques. These skills help reduce mealtime stress and foster a more positive eating environment.	Prasetyo et al., 2017 [93]
**Implementation of Structured Feeding Plans**	**Developing and Monitoring Individualized Feeding Plans:** Nurses collaborate with dietitians to create tailored feeding plans that gradually introduce new foods while ensuring nutritional adequacy. Techniques like food chaining are used to introduce new foods in small, manageable steps.	Białek-Dratwa et al., 2022 [27]
	**Routine Monitoring and Adjustments:** Regular follow-ups are essential to assess progress and adjust feeding plans as necessary. Nurses track the child’s growth, nutritional intake, and psychological well-being to provide comprehensive care.	Richmond et al., 2023 [73]
**Emotional and Social Support**	**Providing Emotional Support:** Nurses offer emotional support by acknowledging the challenges families face and validating their experiences. They provide a listening ear and empathetic responses to alleviate feelings of isolation and frustration.	Oktarina et al., 2023 [92]
	**Facilitating Support Groups:** Nurses can organize or recommend support groups where families share experiences and strategies with others facing similar challenges. These groups provide mutual support and practical advice.	Prasetyo et al., 2017 [93]
**Coordination of Multidisciplinary Care**	**Coordinating Multidisciplinary Care:** Nurses coordinate care among various healthcare providers, including pediatricians, dietitians, psychologists, and gastroenterologists. This ensures comprehensive and integrated care addressing all aspects of ARFID.	Cucinotta et al., 2023 [79]
	**Utilizing Evidence-Based Interventions:** Nurses implement evidence-based interventions and stay updated with the latest research and guidelines in ARFID management. This includes staying informed about new therapeutic techniques and best practices for managing food-related anxiety.	Lee & Wang, 2016 [99]

## Data Availability

No new data were created or analyzed in this study. Data sharing is not applicable to this article.
